# An Alternative Pedagogy for the Teaching of Anatomy and Physiology Beyond the Classroom

**DOI:** 10.15694/mep.2018.0000242.1

**Published:** 2018-10-25

**Authors:** Natalia Bilton, John Rae, Peta Snikeris, Kym Barry, Sarah Milograna, Michelle Reeve, Craig Roberts

**Affiliations:** 1 Charles Sturt University

**Keywords:** Indigenous Pedagogy, Anatomy, Physiology, Teaching, Undergraduate, Alternative

## Abstract

This article was migrated. The article was marked as recommended.

Valuing Indigenous culture is a requirement for teaching at the Charles Sturt University and we enact this on the Port Macquarie and dubbo campuses through the principles of Indigenous pedagogy/andragogy (IP). In this paper we share our experiences of IP-inspired learning activities, in particular our use of the 8 Aboriginal Ways of Learning ( Yunkaporta, 2009), to increase student engagement with anatomy and physiology and support the retention of Indigenous students in their first year of university study. Indeed, we propose that this approach will increase the educational success of *all* students.

## Background

Our students, who are working towards a degree in the Health Sciences, are required to undertake two first-year subjects in anatomy and physiology and this is the context of this paper. We know that our students are likely to vary in their education prior to university ( Anderton, Evans and Chivers, 2016), may feel overwhelmed by these subjects, and that this experience will likely influence their ability to learn key concepts ( Eagleton, 2015). Furthermore, research has shown that our students are also likely to be predominately kinaesthetic learners ( Amir and Subramaniam, 2014) a high proportion of which, also identifying as being either Aboriginal or Torres Strait Islander.

In this context, we propose that three interrelated aspects respond to the characteristics of our students. This is an arrangement of kinesthesis, student involvement and Indigenous Andragogy which is seldom, if ever, referred to in the higher education literature.

The first aim of our work concerns our teaching practice. We would like to share, with other scholars in the field of learning and teaching, an alternative pedagogy/andragogy in the teaching of anatomy and physiology to first year undergraduate students. This student-led kinaesthetic activity can be used to assist in the engagement of students in learning anatomy and physiology and furthermore, we present the potential to incorporate this “hands on” activity into the wider scholarly teaching practice to support student retention.

The second aim of this paper will focus on mapping our teaching practice and resulting activity to three relevant learning theories. The first will be the Learning Cycle which is based on the neuroscience of learning ( Zull, 2002), the second is the 8 Aboriginal Ways of Learning ( Yunkaporta, 2009) and finally with the concept of ‘material thinking’ as it relates to our methods ( Carter, 2004).

## Description of Learning Activity

This learning activity is most appropriate for content that can be described in a two dimensional space. Representations of complex structures and descriptions of stepwise processes are often most suited to this technique. The activity starts with clear communication by the teacher which explains the learning outcome addressed by the activity. The educator then sets out clear guidelines about the kind of image they would like the student to create in order to demonstrate their understanding. Students are then given appropriate materials to use to create their symbols/represent the concept on various surfaces around the campus. Submission of work involves taking a photo and uploading that image to a learning management system. The educator then displays the images submitted (anonymously) in class and discuss the topic, corrects any misconceptions to ensure that students receive timely feedback on their understanding of the concept.

The learning activity described within this paper focuses on the use of symbols and images as both a tool for learning, and a demonstration of understanding. It is designed to encourage students to encode their learning, connect the learning to their own reality and take ownership of the depiction all while sharing their understanding through the drawing of images and symbols outside the traditional learning space ( Zull, 2002; Yunkaporta, 2009). This active learning process aims to encourage deep learning and retention of material in long term memory ( Zull, 2002; Yunkaporta, 2009).

## Digital Showcase

The digital montage, published here https://youtu.be/pCuSTdwUNPs, was created with the assistance of Charles Sturt University’s media technologist for Indigenous resources, Yanhadarramal Jade Flynn. Attention was made when developing this presentation that the content included was culturally relevant and appropriate. Images selected demonstrate several student cohorts engaging with anatomy and physiology content through the use of the IP-inspired learning activity at “our place” on the Port Macquarie campus. This digital montage provides an archive demonstrating the effectiveness of this technique and how it fosters physical engagement with content as well as peer to peer learning. This digital showcase also provides inspiration for potential application of this technique beyond this content area and our place. Substantial evidence is provided in this digital montage that demonstrates students engaging with anatomy and physiology material utilising this chalk technique - in an open area learning environment on campus - that fostered peer learning which shares some similarities with traditional methods used by Indigenous populations to distribute and convey information ( Cameron, 2015).

## The Neuroscience of Learning

The activity reported here provides an opportunity to Anatomy and Physiology students to consolidate learning of concepts discussed in class by actively testing their knowledge, as postulated by neuroscience-based learning theories (Kolb’s, 1939; Zull, 2002). Learning occurs through the progressive construction of neuronal networks upon pre-existent ones, a physical/biological mechanism involving integration among the major areas of the cerebral cortex, which are involved in sensing, integrating and acting on new information. This integration of cerebral areas is based on four processes: (1) experience - sensory and post sensory cortices receive sensory input, achieved through direct information transference from teacher to student - this process is optimised when concrete examples are utilised to access pre-existent neural networks; (2) reflexion - the temporal integrative cortex is involved in thinking about the information just received and remembering it, which may be stimulated for instance by questioning students about the content or demonstrating it in a different manner; (3) abstraction - the frontal integrative cortex is involved in the creation of a cognitive process, elicited when discussing a topic or engaging with a problem solving exercise; and (4) active testing - the premotor and motor cortices are involved in active testing, achieved through motor output (e.g. acting, modifying, creating) (Kolb, 1939; Zull, 2002).

This cyclic mechanism relies on the presence of emotions that facilitate task engagement (e.g. interest), and absence of task-withdrawing emotions, (e.g. distress and lack of confidence in the self). Movement releases dopamine; active learning that involves choice and actions by the learner is pleasurable and effective for developing concepts and applications of knowledge ( Zull, 2002). The chalk activity stimulates students to draw concept maps and diagrams, offering the opportunity to summarise, explain, reframe explanations and think of examples that are familiar to their peers, exercising the motor output phase of the cycle required to consolidate deep learning. Social construction of knowledge is not a new concept (Vygotsky, 1978; Bearison, 1982), and several benefits of peer interaction and feedback have long been reported in the literature as an effective way of learning (King, 2002; Boud et. al., 2014; Nicol et al., 2014) which contributes towards the development of employability skills (Cassidy, 2006). In a study by Nicol et al., (2014) students indicate that peer feedback was useful because the language employed by other students was more understandable and for offering an increase in feedback variety. ‘Peer-assisted and self-directed learning’ encourage graduates to become lifelong learners by managing their own learning, acquiring collaborative and communication skills to articulate knowledge, and to develop ‘reflective practice and critical self-awareness’ (Boud et. al., 2014). The pleasurable motor output achieved when gathering with peers in a safe, friendly environment to exercise teaching and learning using chalk allows students to undergo a metacognitive process that helps them regulating their understanding of new concepts (King, 2002) and building robust synapses ( Zull, 2002).

## Indigenous Pedagogy

Using an Australian Indigenous pedagogical framework for the teaching of anatomy and physiology in the multicultural environment of a mainstream Australian university is often considered fraught with challenges and something that is necessarily a difficult and complex undertaking ( McLoughlin and Oliver, 2000; Aikenhead, 2001; Nakata *et al.*, 2012; Nakata, Nakata and Chin, 2015). At Charles Sturt University, a number of our students reminded us that this need not be the case by using a study strategy that demonstrated instinctive learning processes that we could map to the 8 Aboriginal Ways of Learning ( Yunkaporta, 2009). The 8 Ways present a framework for understanding Indigenous learning in the western nations including the Baakindji, Ngiyampaa, Yuwaalaraay, Gamilaraay, Wiradjuri and Wangkumarra nations, from country in Western New South Wales ( Yunkaporta, 2009; Health, 2014). Here, the learning activity developed by our team, in response to the original student lead creativity proposes a shift in focus from attempting to incorporate Indigenous content into the teaching of anatomy and physiology, to a focus on consciously integrating Indigenous learning processes into our tertiary teaching. As described by Nakata et al. (2012), the objective is not to “decolonise” the content, but rather to change student thought and the learning process.

The 8 Aboriginal Ways of Learning are: story sharing, learning maps, non-verbal learning, symbols and images, land links, non-linear learning, deconstruction and reconstruction as well as community links (for a full understanding of each of the 8 Ways and suggestions for use in teaching, see ( Yunkaporta, 2009; Bat, Kilgariff and Doe, 2014; Health, 2014). It is important to note that a full understanding of the 8 Ways of Learning includes an appreciation of links between each process; Indigenous Ways of Learning are a process that require use of numerous ways of learning in order to reach knowing ( Yunkaporta, 2009; Bat, Kilgariff and Doe, 2014). Notwithstanding the idea that all learning comes from this combination of learning processes, the activity we developed is specifically discussed below in terms of: symbols and images, and community links, including comment on how these 8 Aboriginal Ways of Learning interface with Western pedagogical models.

Further consideration of the neuroscience research on learning, demonstrates an association between emotion and deep learning ( Zull, 2002; Yunkaporta, 2009). The 8 Aboriginal Ways of Learning emphasizes learning through, and for, community, signifying that learning should be centred on relationships and local viewpoints ( Yunkaporta, 2009; Bat, Kilgariff and Doe, 2014). The purpose is to tie learning into systems of relationships and bring new knowledge back to support your community. As described in this paper, this was something students instinctively tapped into by drawing in common areas of the campus, and working together to both develop and share their knowledge within their community, and outside. Thus, strengthening relationship networks both within immediate class or discipline groups, and across the wider university community should result in enhanced learning according to both Indigenous and western pedagogical models ( Yunkaporta, 2009; Zull, 2002).

Remembering that the 8 Aboriginal Ways of Learning represents a model of some general Indigenous pedagogies, not a program to map against teaching plans ( Yunkaporta, 2009; Bat, Kilgariff and Doe, 2014; Health, 2014); and although we have discussed the teaching pedagogy we are presenting in relation to these theories, the intention is to highlight the cultural inclusiveness of a spontaneous student activity. We do not presume to own this approach or to prescribe it as either universal or local. The paradigm represented by the 8 Ways aims to be supportive of cultural inclusion and embedding culture in teaching is certainly not something to be trivialised ( Yunkaporta, 2009; Bat, Kilgariff and Doe, 2014; Health, 2014). Embedding culture into our teaching can be daunting for many of us who may not feel as competent as we would like and while cultural inclusiveness is not something to be underestimated I hope we have shown that adding culture to learning need not always be content based, but can be woven into the very learning processes we use.

## Material Thinking

Fundamental to the 8 Aboriginal Ways of Learning ( Yunkaporta, 2009) are things of a material nature. You will have noted on the video the chalk, concrete, marks on concrete, and glass, symbols, representations of the respiratory system, cellular processes, and so forth. One might extend this list to other ‘Entities in the land like stones, animals, plants and rivers [which] all [provide] knowledge’ ( Yunkaporta, 2009). Indeed, these things provide ‘deep’ knowledge, says Yunkaporta (2009). Paul Carter (2004, p. XI) used the term ‘material thinking’ to describe this sort of thing - ‘an intellectual adventure’, he called it. It is a way of thinking that is ‘peculiar to the making process’ ( Carter, 2004)- making drawings, as you saw in the video.

We can take this argument about materials and material thinking one step further - to the concept of ‘place’. We agree with what Lauren Vaughan, the design scholar. She said she was ‘concerned with the individual, their experiences, and their sense of placed self’ ( Vaughan, 2008). Material thinking helps to extend our perspective from students to students in relation to place - their place, and Yunkaporta says: we should ‘link content back to land and place’. It works in the opposite direction too: ‘the materialising of ideas through materials and processes, is a process of place-making’ ( Vaughan, 2008).

To think more globally - Margaret Somerville, an education scholar, says that place connects the local and the global ( Somerville, 2008). Somerville explains that without an intimate knowledge of local places that we love there is no beginning point. That is, without a concept of the local, action is not possible. Under conditions of global contemporaneity, it is no longer possible to consider local issues, such as health or education, as independent of global issues. Knowing one’s ‘place’ is important.

## Conclusions

Our work demonstrates that implementing reflective practice into higher education need not be complicated. The Level-3 teacher, as defined by ( Biggs, 2011), is one who constantly enquires him/herself what actions performed by their students are more likely to entail ‘deep’ learning of key concepts, favouring the constructive alignment of subjects. Reflecting upon in which ways a practice represents current pedagogies may support the establishment of new strategies that are grounded on scientifically validated methods. Here, we describe and reflect on a simple and accessible student-led learning strategy which stimulates active testing, a frequently overlooked phase of Zull’s learning cycle ( Zull, 2002), in higher education. This strategy also incorporates material thinking and both Indigenous and Western pedagogical frameworks, to address the learning needs of our diverse student cohort.

## Take Home Messages

• This study demonstrates how easy and enjoyable good teaching practice in higher education can be, and that implementing reflective practice into higher education teaching doesn’t need to be complicated.
• The recording of digital montages can be useful artifacts contributed to the learning and teaching community.
• Learning activities and teaching practise can be driven by neuoscience, Indigenous and arts-based pedagogical foundations.

## Notes On Contributors

Natalia Bilton received her PhD from the School of Behavioural Sciences at the University of Newcastle. After her post-doc, she moved to Thursday Island, Queensland, where she taught Torres Strait Islander students at James Cook University. Next, she was involved in teaching neuroscience in the School of Medicine and Dentistry in Townsville. In 2013, she joined the School of Biomedical Sciences, at Charles Sturt University. Natalia’s research interests include creativity, neuroscience and Indigenous pedagogy in anatomy and physiology.

John Rae is a Senior Lecturer in Health Services Management. He teaches in the School’s programs in health service management. Originally from a nursing background, John has worked for 35 years in the public health system in varying roles including health planning, service development, aged care management, quality management, project management, capital works redevelopment, genetic counselling, genetic counselling education, nurse education and clinical nursing.An interest in creativity prompted John to complete a PhD at CSU where he examined the place of creativity in public health services. He continues to work in this and related areas.

Peta Snikeris currently works at the School of Community Health, Charles Sturt University. Here, Peta thoroughly enjoys teaching the first year physiology subjects of BMS161 and BMS162 (Health and the Human Body Parts I and II). In addition, Peta is part of the teaching team for Pharmacology and Neuroscience. Peta’s research prior to joining CSU focused on Neuroscience, Molecular Biology and Immunology.

Kym Barry commenced work as Lecturer in Medical Radiation Sciences - Nuclear Medicine in 2016 at the Port Macquarie campus. Kym graduated from Newcastle University with Bachelor of Applied Science - Medical Radiation Technology in 1997. Kym completed Master of Medical Radiation Science - Nuclear Medicine at Charles Sturt University in 2015. Kym trained at St Vincent’s Hospital in Sydney. Kym has over 17 years clinical experience in Nuclear Medicine in a variety of clinical settings including large and rural public hospitals and also private companies.

Dr Sarah Milograna is a Bachelor of Biological Sciences graduated from the University of Sao Paulo (Brazil) in 2008. From the same University, she obtained a Master of Science (2010), and a PhD in Science (2015). She is teaching Human Biosciences for first-year nursing students at Charles Sturt University since 2017.

Michelle Reeve joined CSU at the beginning of 2016 as a Lecturer in Medical Radiation Science - Medical Imaging, after working in the clinical environment for the last 13 years. Before joining CSU, Michelle had a passion for undergraduate clinical education of medical imaging students, being a Clinical Supervisor at a large tertiary referral hospital in Western NSW. Michelle graduated from the University of Newcastle in 2002 with a Bachelor of Medical Radiation Science (Diagnostic Radiography) - With Merit. She completed her Master of Medical Radiation Science - Radiographic Image Interpretation at CSU in July 2016.

Mr Craig Roberts graduated from Charles Sturt University with a Bachelor in Applied Science (Medical Imaging) in 1998. He has worked clinically as a radiographer until 2017 specialising in computed tomography (CT) and magnetic resonance imaging (MRI), holding senior clinical roles in addition to acting as a clinical supervisor for undergraduate students. In 2017, Craig commenced work as an associate lecturer in Medical Radiation Science, specialising in clinical CT.
