# Supplementary material for: An Alternative Pedagogy for the Teaching of Anatomy and Physiology Beyond the Classroom
Source: MedEdPublish (2016). 2018 Oct 25;7:242. [Version 1] doi: 10.15694/mep.2018.0000242.1 (PMC10711945; doi:10.15694/mep.2018.0000242.1)
Supplement: Peer review history

# Reviewer report: Reviewer response for version 1

**DOI:** 10.21956/mep.19682.r29404

**Published:** 2019-06-28

**Authors:** Iain Keenan

**Affiliations:** 1 Newcastle University

This review has been migrated. The reviewer awarded 2 stars out of 5 This is an interesting article addressing the importance of identifying and embracing the diversity of learners and while effectively facilitating the education of all students. This paper provides some useful practical strategies for doing so.However, I do have some reservations. First of all, the authors mention kinaesthetic learning. VARK and other learning styles models have been effectively discredited (Coffield, 2004; Hall, 2016), and therefore should not be used as a basis for implementing educational approaches. In this context, suggesting that certain populations of students have particular learning styles without providing strong supporting evidence does not suggest a sufficiently scholarly and robust approach has been employed by the authors. It should be noted that the reference for Kolb with respect to the experiential learning cycle should be (Kolb, 1984), rather than (Kolb, 1939). This citation also does not appear in the reference list. It should be noted that while (Zull, 2002) used Kolb’s learning cycle as the basis for some of his ideas in his book, that this does not mean the learning cycle in based on neuroscience. It should also be noted that Zull’s book is popular non-fiction which has not been peer-reviewed, as opposed to a scholarly work. I therefore have reservations about the authors relying heavily on (Zull, 2002) to support the concepts described. As the authors state in their conclusion that arts-based pedagogical foundations can support learning activities, I would like to have seen further exploration of the basis and findings relating to artistic learning methods. For example, our paper (Backhouse, Fitzpatrick, Hutchinson, Thandi, & Keenan, 2017), utilised Kolb’s learning cycle as a basis for the design of an artistic learning approach in anatomy educationReferences are provided below for interest. Coffield, F., Moseley, D., Hall, E. Ecclestone, K. (2004). Learning Styles and Pedagogy in post-16 learning: a systematic and critical review. London, England: Learning and Skills Research Centre.Hall, E. (2016). The tenacity of learning styles: a response to Lodge, Hansen, and Cottrell. Learning: Research and Practice, 2(1), 18-26. doi:10.1080/23735082.2016.1139856Kolb, D. A. (1984). Experiential learning : experience as the source of learning and development. Englewood Cliffs, N.J.: Prentice-Hall.

Reviewer Expertise:

NA

No decision status is available

# Reviewer report: Reviewer response for version 1

**DOI:** 10.21956/mep.19682.r29405

**Published:** 2018-10-26

**Authors:** Richard Hays

**Affiliations:** 1 James Cook University

This review has been migrated. The reviewer awarded 4 stars out of 5 As an Australian medical educator, I really enjoyed reading about strategies that aim to engage more effectively with biomedical science students from our Indigenous communities. Similar work has been done with First Nations peoples in North America, and it would be interesting to see if the Australian approach is as effective. That is the small piece that foe me is missing - an evaluation of impact on enrolments, attrition rates, assessment results. That would earn an extra star. As an international medical educator, I was struck by how the authors have implemented flexible, innovative strategies that may be more widely popular and successful. Anatomy is a favourite subject of many medical students (mine too!) and yet I was taught anatomy in much the same, teacher-centric way as my predecessors during the last 400 years. The reduction in face-2-face dissection-based and didactic teaching is actually an opportunity to do things much better, utilising the amazing resources on the web, the power of art and sculpture, body painting, playing (yes, playing) with models, and small group activities that involve drawing, peer learning and discussion. Perhaps we can all learn from Indigenous cultures how to bring a curriculum to life?

Reviewer Expertise:

NA

No decision status is available

# Reviewer report: Reviewer response for version 1

**DOI:** 10.21956/mep.19682.r29406

**Published:** 2018-10-25

**Authors:** P Ravi Shankar

**Affiliations:** 1 American International Medical University

This review has been migrated. The reviewer awarded 5 stars out of 5 I enjoyed reading this article and watching the YouTube video. Creating drawings using chalk and other media has been employed by various indigenous cultures all over the world. The authors have provided a vivid description of the student-led kinesthetic activity and the neuroscience of learning. The video explained the activity very well. Our increasing knowledge about the neuroscience of learning is being employed to make student learning more efficient. Linking emotions with learning is an effective strategy and one which knew intuitively, long before it was shown by science. We remember a gripping story much more clearly than boring sets of numbers. The eight Aboriginal ways of learning were new to me though tribes in other locations do use similar strategies. The concept of material thinking and the concept of place have been explained well. I found this article interesting and thought provoking and would recommend it to all medical educators.

Reviewer Expertise:

NA

No decision status is available
